# Comparative evaluation of molecular technologies for the identification of prevalent non-tuberculous mycobacteria in pulmonary infections: a systematic review and meta-analysis

**DOI:** 10.1080/07853890.2026.2626123

**Published:** 2026-02-10

**Authors:** Xiang Chen, Jiawen Sun, Xibin Chen, Yanshan You, Leonardo Antonio Sechi, Paola Molicotti

**Affiliations:** a Health Care Center, The First Affiliated Hospital of Shantou University Medical College, Shantou, Guangdong, China; b Department of Biomedical Sciences, University of Sassari, Sassari, Italy; c Department of Gastroenterology, Shenzhen Guangming District People’s Hospital, Shenzhen, Guangdong, China; d Shantou Polytechnic, Shantou, Guangdong, China; e Department of Education, Shantou Tuobin Vocational Technical School, Shantou, Guangdong, China; f SC Microbiologia, AOU Sassari, Sassari, Italy

**Keywords:** Non-Tuberculous Mycobacteria (NTM) identification, NTM pulmonary disease, molecular technologies, performance evaluation, antibiotic resistance

## Abstract

**Background:**

The increasing prevalence of non-tuberculous mycobacteria pulmonary disease (NTM PD) is a burden to public health. Successful management of NTM PD critically depends on accurate species identification and reliable drug susceptibility testing to guide appropriate antibiotic therapy. Emerging molecular technologies offer rapid diagnostic solutions compared to conventional methods, but their performance varies. This study aims to provide a comprehensive evaluation of current molecular techniques for NTM identification and to present a global antibiotic resistance profile.

**Methods:**

A systematic literature search was conducted in PubMed and Web of Science for studies published between 2005 and 2024. Studies applying molecular methods for NTM identification and resistance detection in humans were included. Data on study characteristics, diagnostic methods, sample types, sample sizes, identification sensitivity, and drug susceptibility results were extracted. Meta-analysis was performed using R with the *meta4diag* package. The quality of included studies was assessed using the QUADAS-2 tool.

**Results:**

The analysis included 49 studies on NTM identification and 33 studies on antibiotic resistance. For species identification, all evaluated molecular technologies (MALDI-TOF MS, PCR-based methods, Sequencing, DNA chip, and DNA strip) demonstrated high pooled sensitivities (>0.92). Subgroup analysis revealed that sample type significantly affected performance for MALDI-TOF MS. Preliminary analysis of antibiotic resistance rates revealed varying patterns. For slowly growing mycobacteria, a significantly high Ethambutol resistance rate was observed in *M. avium* (69.20%). Among rapidly growing mycobacteria, resistance to Imipenem was notable (54.22%), and Clarithromycin resistance varied significantly within the Mycobacterium abscessus complex.

**Conclusion:**

Emerging molecular technologies have revolutionized the methodology for NTM identification with excellent performance. However, their performance can be influenced by sample type, particularly for MALDI-TOF MS. The alarming and heterogeneous antibiotic resistance patterns also highlight the critical need for rapid and accurate species identification and drug susceptibility testing to inform effective therapeutic strategies.

**Key messages**
1. Molecular technologies demonstrate high accuracy for NTM identification.
2. Antibiotic resistance is a serious concern with variations among NTM species and subspecies.
3. Rapid and accurate species identification and drug susceptibility testing are crucial for guiding effective clinical management of NTM PD.

## Introduction

Non-Tuberculous Mycobacteria (NTM), a group of mycobacteria other than Tuberculosis and *Mycobacterium Leprae*, consists of over 190 species and 14 subspecies [1]. Conventional wisdom holds that most of the NTMs are opportunistic pathogens and cause infection only under certain circumstances, such as in individuals with underlying lung diseases or immune suppression. However, research reported that the morbidity of NTM pulmonary disease had an annual increase of 8.2% in the USA since the 1990s and 6.5% in Canada since 1998 [2,3]. Not just in North America, similar trends have been observed in Asia and Europe [4–8]. The increasing prevalence of NTM Pulmonary Disease (NTM PD) in these countries has been appreciated. As for the treatment of NTM PD, a regimen based on precise species identification and drug susceptibility testing is highly recommended according to the Official ATS/ERS/ESCMID/IDSA Clinical Practice Guideline [9]. The severity of NTM infection is different among species. Patients with *Mycobacterium intracellulare* infection had more severe clinical manifestations and worse prognosis than those with *Mycobacterium avium* infection [10]. Besides, *Mycobacterium chimaera*, a subspecies of *M. intracellulare* which was usually considered less likely to cause lung disease than other strains of Mycobacterium Avium Complex (MAC), might lead to an even worse clinical outcome in specific infection caused by contaminated medical heater-cooler units [11]. Furthermore, the therapeutic efficacy of antibiotics also varies from species.

Over the last two decades, emerging molecular biotechnologies have revolutionized the methodology for identifying pathogenic microorganisms. Specific to NTM and its antibiotic resistance, identification techniques were limited to biochemical tests, culturing, and acid-fast based microscopy smear examination in the early twenty-first century. The accuracy and timeliness were highly dependent on the lab technician. Nowadays, with the application of Matrix-Assisted Laser Desorption Ionization–Time of Flight Mass Spectrometry (MALDI-TOF MS), Polymerase Chain Reaction (PCR) based techniques, Sequencing, DNA chip, and DNA strip, the identification accuracy and efficiency have been significantly improved [12–16]. Up to the present, no study has conducted a systematic comparison among technologies such as MALDI, PCR, Sequencing, DNA chip, DNA strip, and others. Hence, this study aims to evaluate the performance of these techniques in identifying NTM species or subspecies, and to analyse the effect of testing conditions on their efficacy. In addition, we present the global antibiotic resistance situation of NTM.

## Methods

### Search strategy and selection criteria

The conduct of this study is based on the Preferred Reporting Items for Systematic reviews and Meta-Analyses (PRISMA) guidelines. A comprehensive literature search was performed on PubMed and Web of Science with the following keywords: ((non-tuberculous mycobacteria) OR ((mycobacterium) AND ((avium) OR (chimaera) OR (intracellulare) OR (kansasii) OR (xenopi) OR (abscessus))) AND (identification) NOT ((review) OR (comment) OR (meta-analysis)). The latest literature search was conducted on December 31, 2024. The range of publication dates was set at the last twenty years. All original studies on the identification of pulmonary NTM infections and antibiotic resistance using emerging molecular technologies were considered for inclusion. Studies would be excluded if they met the following criteria: 1) Studies without an English version available; 2) Animal or environmental research on NTM; 3) Studies reported Non-pulmonary NTM infection; 4) Studies without a full-text version available; 5) Unrelated search records. A review of article titles, abstracts and full-text was performed by two authors (X.C., J.S.) independently after the removal of duplicate records.

### Data extraction

For all the included studies, information on characteristics (Author name, Study name, Publication year, and Study region) was extracted. For studies on NTM identification, data on methods, sample type, sample size, overall sensitivity, and individual sensitivity for species or subspecies were extracted. For studies on Antibiotic resistance, the drug susceptibility results of Amikacin, Clarithromycin, and Moxifloxacin were extracted. The mark “-” in the table represents data not available. All data were checked by two authors (X.C., J.S.).

### Quality assessment of included studies

The risk of bias of all enrolled studies was assessed by two authors (Y.Y., X.C.) using the tool for Quality Assessment of Diagnostic Accuracy Studies (QUADAS-2). QUADAS-2 considers four domains (11 questions in total), including: 1) Patient Selection, 2) Index Test, 3) Reference Standard, and 4) Flow and Timing. With “Yes” answers to all eleven questions, the included study will be ranked as low risk of bias. With one or more “Unclear” and “No” answers, it will be ranked separately as moderate and high risk of bias. Disagreements between these two authors would be resolved by the third author.

### Statistical analysis and data visualization

SPSS Statistics (version 26.0, IBM Corporation, New York, USA) was applied for statistical analysis. The meta-analysis data synthesis and visualization were performed in R with the *meta4diag* and *robvis* packages (version 4.5.0, The University of Auckland, New Zealand), GraphPad Prism (version 10.3.1, Dotmatics, Boston, USA) and Adobe Illustrator (version 26.2.1, Adobe Inc., San Jose, USA).

## Results

### Study selection

**Figure 1.**
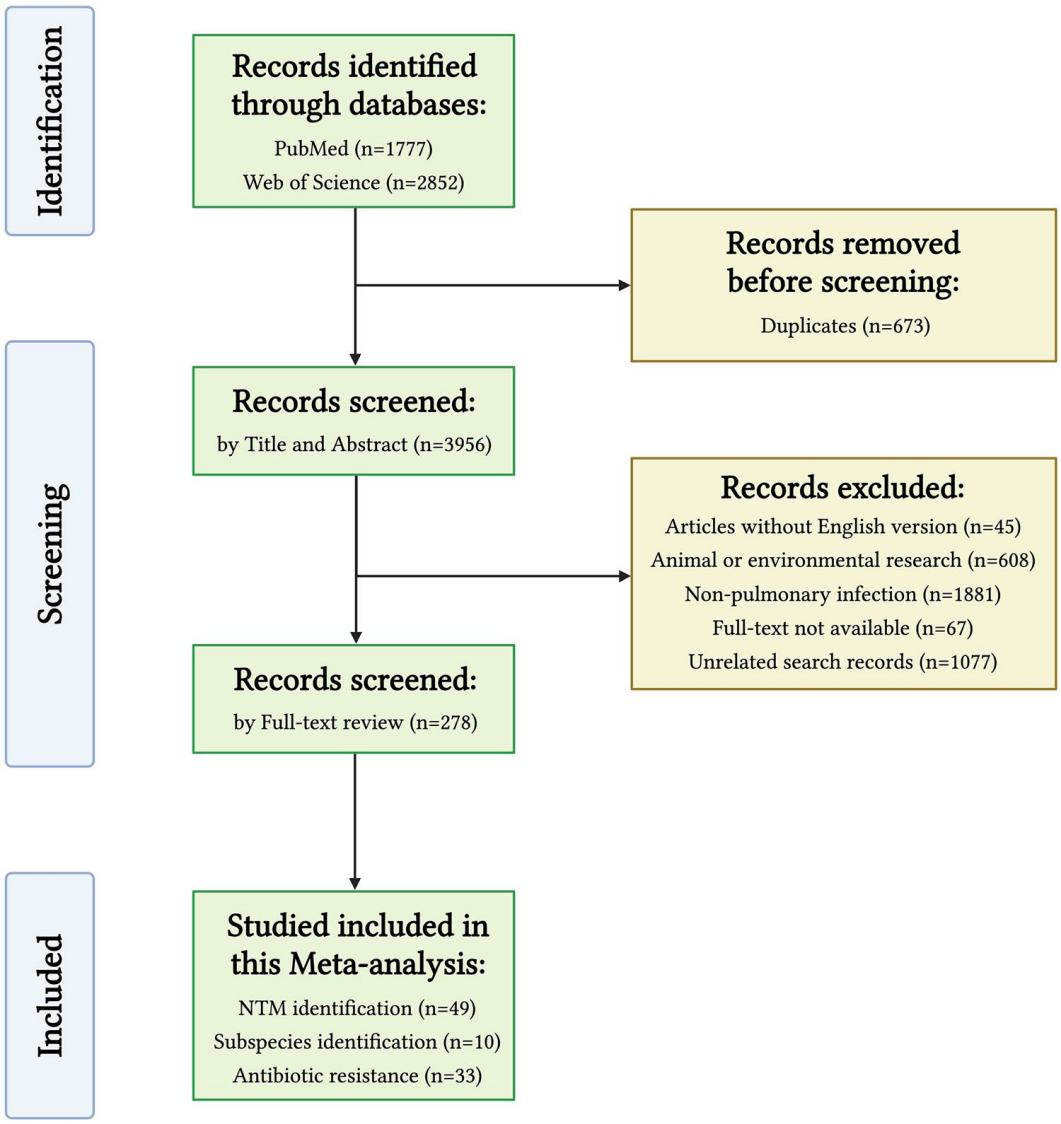
Flowchart of study selection.

The literature search found 4629 publications, 1777 records from PubMed and 2852 records from Web of Science. After the duplicates removal (*n* = 673), 3956 records continue with the screening by title and abstract. In this step, 45 records without an English version, 608 records of animal or environmental research, 1881 records of non-pulmonary infection, 67 records without full-text available, and 1077 unrelated search records were excluded. Finally, 49 studies on NTM identification [17–65], 10 studies on subspecies identification [66–75], and 33 studies on antibiotic resistance [53,72,75–105], were included in this meta-analysis (Figure 1). Among the 33 antibiotic resistance studies, three records are duplicated in the identification part.

### Study quality assessment

**Figure 2.**
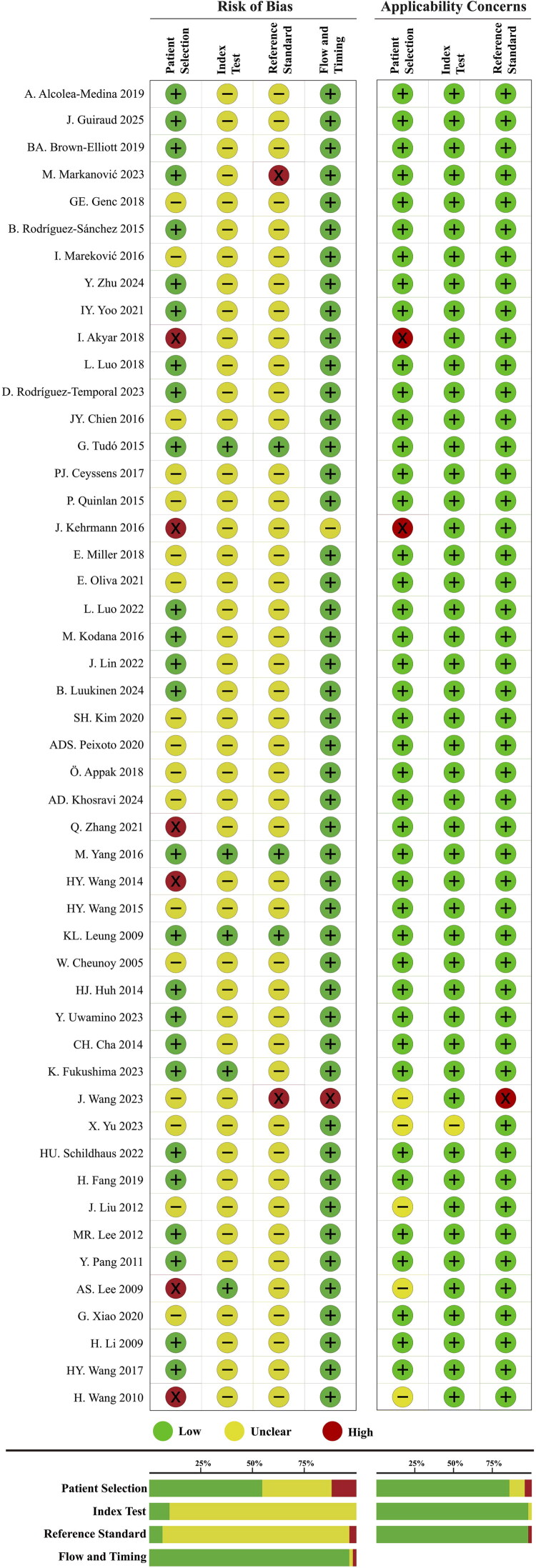
Quality assessment of NTM species identification studies.

**Figure 3.**
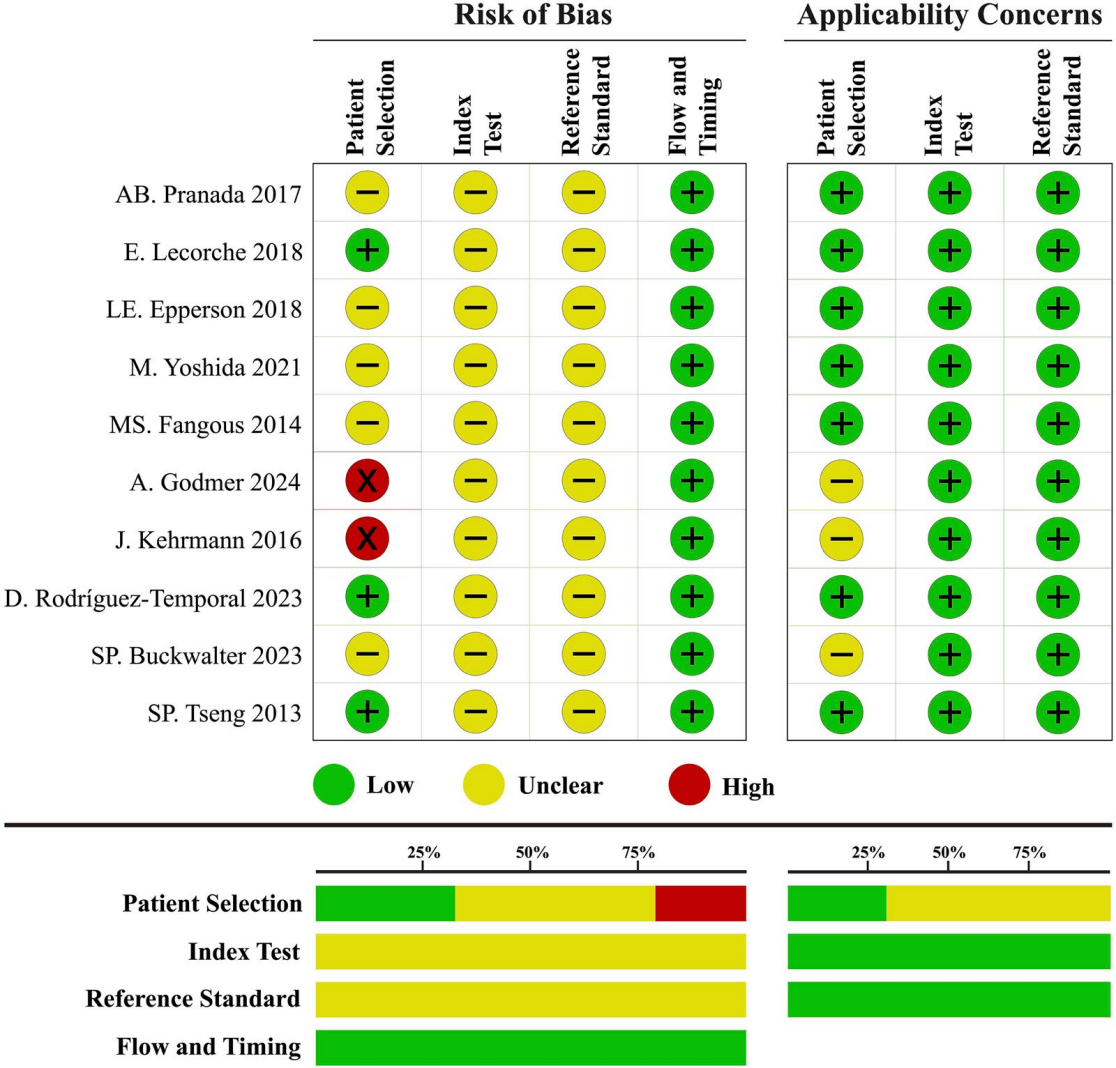
Quality assessment of NTM subspecies identification studies.

The majority of the NTM species identification studies (*n* = 38, 77.55%) were ranked as moderate risk of bias. For the remaining 11 studies, 3 were ranked as low risk of bias (6.12%), and 8 were high risk of bias (16.33%). About the subspecies identification studies, 2 were ranked as high risk of bias (20%), and 8 were moderate risk (80%). The risk of bias mainly came from inappropriate exclusions of patient selection and the blind interpretation between the index test and reference standard (Figures 2 and 3). The details of eleven questions and answers for these four domains are presented in Supplementary File 1.

### Evaluation of NTM identification technologies

**Figure 4.**
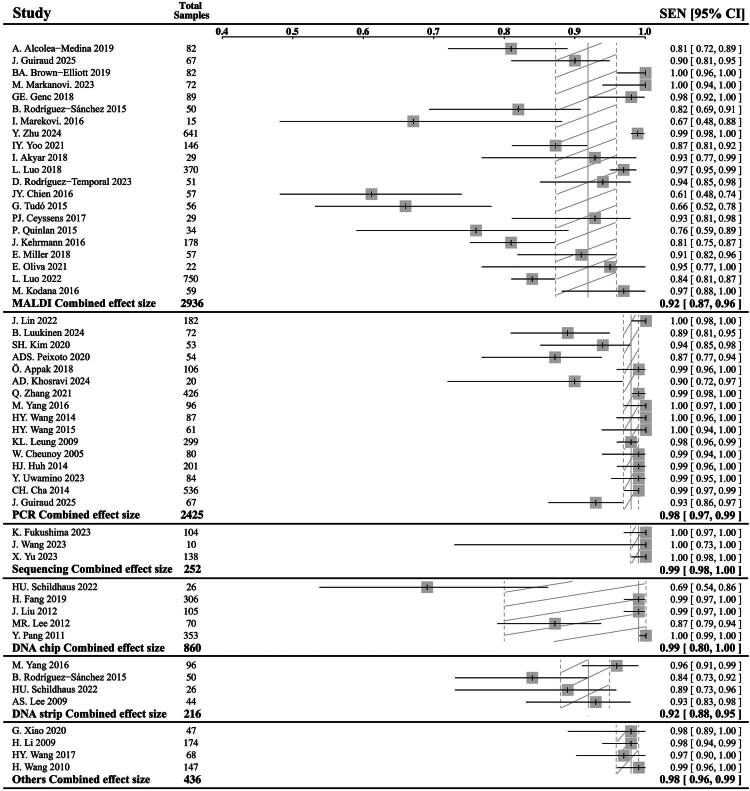
Forest plot of NTM species identification studies.

In the 49 NTM species identification studies, 4 studies applied two technologies at the same time [18,22,45,56]. The characteristics of the studies were presented in Table 1. Overall, MALDI was the most commonly used and applied in 21 studies. The second was PCR-based techniques, applied in 16 studies. There were 3 studies with the application of the sequencing-based method, 5 with DNA chip, 4 with DNA strip and 4 with other techniques. As shown in Figure 4, with a total sample of 2936, the MALDI studies had a combined effect size of 0.92 in sensitivity (95% CI: 0.87, 0.96). The PCR-based studies had a total of 2425 samples and a synthetic sensitivity of 0.98 (95% CI: 0.97, 0.99). The sequencing-based studies had a total of 252 samples and a synthetic sensitivity of 0.99 (95% CI: 0.98, 1.00). The DNA chip studies had a total of 860 samples and a synthetic sensitivity of 0.99 (95% CI: 0.80, 1.00). The DNA strip studies had a total of 216 samples and a synthetic sensitivity of 0.92 (95% CI: 0.88, 0.95). Studies applying other techniques had a total of 436 samples and a synthetic sensitivity of 0.98 (95% CI: 0.96, 0.99). Details of the individual sensitivity for identifying six pathogens that cause NTM PD and a summary of each identification method were available in Supplementary Files 2 and 3.

**Table 1.** Characteristics of NTM species identification studies.

Study	Region	Year	Method	Sample type	Total sample No. & Sensitivity	Overall risk of bias
A. Alcolea-Medina et al.	UK	2019	MALDI-TOF	Solid medium	82 (0.805)	Moderate
J. Guiraud et al.	France	2025	MALDI-TOF	Solid medium	19 (1.000)	Moderate
				Liquid medium	48 (0.854)	
BA. Brown-Elliott et al.	USA	2019	MALDI-TOF	Solid medium	82 (1.000)	Moderate
M. Markanović et al.	Croatia	2023	MALDI-TOF	Liquid medium	72 (1.000)	High
GE. Genc et al.	Turkey	2018	MALDI-TOF	Liquid medium	89 (0.978)	Moderate
B. Rodríguez-Sánchez et al.	Spain	2015	MALDI-TOF	Solid medium	50 (0.820)	Moderate
I Mareković et al.	Croatia	2016	MALDI-TOF	Liquid medium	15 (0.667)	Moderate
Y. Zhu et al.	China	2024	MALDI-TOF	Solid medium	641 (0.989)	Moderate
IY. Yoo et al.	Korea	2021	MALDI-TOF	Liquid medium	146 (0.870)	Moderate
				Subculture	146 (0.959)	
I. Akyar et al.	Turkey	2018	MALDI-TOF	Solid medium	29 (0.931)	High
L. Luo et al.	China	2018	MALDI-TOF	Solid medium	370 (0.973)	Moderate
D. Rodríguez-Temporal et al.	Spain	2022	MALDI-TOF	Liquid medium	51 (0.941)	Moderate
JY. Chien et al.	Korea	2016	MALDI-TOF	Liquid medium	57 (0.614)	Moderate
G. Tudó et al.	Spain	2015	MALDI-TOF	Solid medium	32 (0.688)	Low
				Liquid medium	24 (0.625)	
PJ. Ceyssens et al.	Belgium	2016	MALDI-TOF	Solid medium	29 (0.931)	Moderate
P. Quinlan et al.	Ireland	2014	MALDI-TOF	Solid medium	11 (1.000)	Moderate
				Liquid medium	23 (0.652)	
J. Kehrmann et al.	Germany	2015	MALDI-TOF	Solid medium	89 (0.775)	High
				Liquid medium	89 (0.843)	
E. Miller et al.	USA	2018	MALDI-TOF	Solid medium	29 (0.931)	Moderate
				Liquid medium	28 (0.893)	
E. Oliva et al.	Italy	2021	MALDI-TOF	Solid medium	11 (1.000)	Moderate
				Liquid medium	11 (0.909)	
L. Luo et al.	China	2022	MALDI-TOF	Liquid medium	750 (0.839)	Moderate
M. Kodana et al.	Japan	2015	MALDI-TOF	Liquid medium	59 (0.966)	Moderate
J. Lin et al.	China	2022	PCR	Liquid medium	182 (1.000)	Moderate
B. Luukinen et al.	Finland	2024	PCR	Solid medium	21 (0.905)	Moderate
				Liquid medium	51 (0.882)	
SH. Kim et al.	Korea	2020	PCR	Undeclared	53 (0.943)	Moderate
ADS. Peixoto et al.	Brazil	2020	PCR	Solid medium	54 (0.870)	Moderate
Ö. Appak et al.	Turkey	2018	PCR	Solid medium	106 (0.991)	Moderate
AD. Khosravi et al.	Iran	2024	PCR	Solid medium	20 (0.900)	Moderate
Q. Zhang et al.	China	2021	PCR	Clinical specimen	426 (0.991)	High
M. Yang et al.	Korea	2016	PCR	Solid & Liquid medium	96 (1.000)	Low
HY. Wang et al.	Korea	2014	PCR	Clinical specimen	87 (1.000)	High
HY. Wang et al.	Korea	2015	PCR	Liquid medium	61 (1.000)	Moderate
KL. Leung et al.	China HK	2009	PCR	Solid medium	299 (0.980)	Low
W. Cheunoy et al.	Thailand	2005	PCR	Solid medium	80 (0.988)	Moderate
HJ. Huh et al.	Korea	2014	PCR	Solid & Liquid medium	201 (0.990)	Moderate
Y. Uwamino et al.	Japan	2023	PCR	Clinical specimen	84 (0.964)	Moderate
				Solid medium	84 (1.000)	
CH. Cha et al.	Korea	2014	PCR	Solid & Liquid medium	536 (0.987)	Moderate
J. Guiraud et al.	France	2025	PCR	Solid medium	19 (1.000)	Moderate
				Liquid medium	48 (0.896)	
K. Fukushima et al.	Japan	2023	Sequencing	Liquid medium	104 (1.000)	Moderate
J. Wang et al.	China	2023	Sequencing	Clinical specimen	10 (1.000)	High
X. Yu et al.	China	2023	Sequencing	Solid medium	138 (1.000)	Moderate
HU. Schildhaus et al.	Germany	2022	DNA chip	Clinical specimen	26 (0.692)	Moderate
H. Fang et al.	China	2019	DNA chip	Solid medium	306 (0.987)	Moderate
J. Liu et al.	China	2012	DNA chip	Solid medium	105 (1.000)	Moderate
MR. Lee et al.	China TW	2012	DNA chip	Solid & Liquid medium	70 (0.871)	Moderate
Y. Pang et al.	China	2011	DNA chip	Solid medium	353 (1.000)	Moderate
M. Yang et al.	Korea	2016	DNA strip	Solid & Liquid medium	96 (0.958)	Low
B. Rodríguez-Sánchez et al.	Spain	2015	DNA strip	Solid medium	50 (0.840)	Moderate
HU. Schildhaus et al.	Germany	2022	DNA strip	Clinical specimen	26 (0.885)	Moderate
AS. Lee et al.	Australia	2009	DNA strip	Solid medium	44 (0.932)	High
G. Xiao et al.	China	2020	Cas12a/gRNA	Liquid medium	47 (0.979)	Moderate
H. Li et al.	USA	2009	Flow cytometry	Liquid medium	174 (0.977)	Moderate
HY. Wang et al.	Korea	2017	Quantamatrix	Solid & Liquid medium	68 (0.971)	Moderate
H. Wang et al.	China	2010	SNP genotyping	Solid medium	147 (0.986)	High

**Table 2.** Comparison of identification sensitivity by sample types.

Method	Solid medium	Liquid medium	Clinical specimen
MALDI-TOF	0.958 (1289/1345)	0.852 (1246/1462)	–
PCR	0.950 (771/812)	0.968 (331/342)	0.988 (590/597)
Others	0.985 (1126/1143)	0.985 (320/325)	0.823 (51/62)
Summary	0.965 (3186/3300)	0.891 (1897/2129)	0.973 (641/659)

A subgroup analysis comparing the identification sensitivity among different sample types was conducted. Studies that did not clearly state the sample type or separate the results from different sample types would be excluded in this part. As shown, the overall sensitivity of solid medium, liquid medium and clinical specimen was 0.965, 0.891, and 0.973, respectively. Sample types strongly influence the efficacy of MALDI-TOF. The sensitivity of the colony from solid medium (0.958) is significantly higher than the liquid medium (0.852) (*p* < 0.01). For other methods, the sensitivity of DNA chip was also significantly lower when using clinical specimens (0.692). However, different sample types would not affect the performance of PCR, Sequencing and DNA strip.

### NTM subspecies identification

In this part, studies focused on distinguishing *M. intracellulare* subspecies *chimaera* from *M. intracellulare* (*n* = 3) and *M. abscessus* subspecies *bolletii, massiliense* from *M. abscessus* (*n* = 7) were included. The characteristics of these 10 studies were presented in Tables 3 and 4. For subspecies identification, MALDI-TOF remains the favourite choice for researchers, applied in 6 studies. With user-customized algorithms or machine learning, MALDI-TOF was able to identify NTM at the subspecies level; however, its efficacy is still dependent on the sample type. Additionally, sequencing-based technique continues to excel in subspecies identification, with only 3 false negatives in 100 samples.

**Table 3.** Characteristics of *M. intracellulare* subspecies identification studies.

Study	Region	Year	Method	Sample type	Total sample No. & Sensitivity		Overall risk of bias
					*M. chimaera*	*M. intracellulare*	
AB. Pranada et al.	Germany	2017	MALDI-TOF	Solid & Liquid medium	45 (0.831)	14 (1.000)	Moderate
E. Lecorche et al.	France	2018	Sequencing	Solid medium	28 (0.964)	8 (0.875)	Moderate
			DNA strip		28 (1.000)	8 (0.875)	
			Line probe		28 (1.000)	8 (0.750)	
LE. Epperson et al.	Germany	2018	MALDI-TOF	Solid medium	55 (0.745)	56 (0.982)	Moderate

**Table 4.** Characteristics of *M. abscessus* subspecies identification studies.

Study	Region	Year	Method	Sample type	Total sample No. & Sensitivity			Overall risk of bias
					*M. abscessus*	*M. bolletii*	*M. massiliense*	
M. Yoshida et al.	Japan	2021	PCR	Solid medium	92 (0.989)	4 (1.000)	53 (1.000)	Moderate
			DNA probe		92 (1.000)	4 (1.000)	53 (0.981)	
MS. Fangous et al.	France	2014	MALDI-TOF	Solid medium	19 (1.000)	14 (1.000)	16 (0.813)	Moderate
A. Godmer et al.	France	2024	MALDI-TOF	Solid medium	119 (1.000)	24 (1.000)	72 (1.000)	High
				Liquid medium	101 (0.980)	20 (0.200)	66 (0.636)	
J. Kehrmann et al.	Germany	2016	DNA strip	Liquid medium	28 (0.929)	3 (1.000)	19 (0.895)	High
D. Rodríguez-Temporal et al.	Spain	2023	MALDI-TOF	Solid medium	157 (0.973)	52 (0.917)	116 (0.781)	Moderate
SP. Buckwalter et al.	USA	2023	Sequencing	Undeclared	36 (1.000)	4 (0.750)	24 (1.000)	Moderate
SP. Tseng et al.	China TW	2013	MALDI-TOF	Solid medium	64 (1.000)	–	64 (1.000)	Moderate

### Preliminary analysis of antibiotic resistance patterns

**Figure 5.**
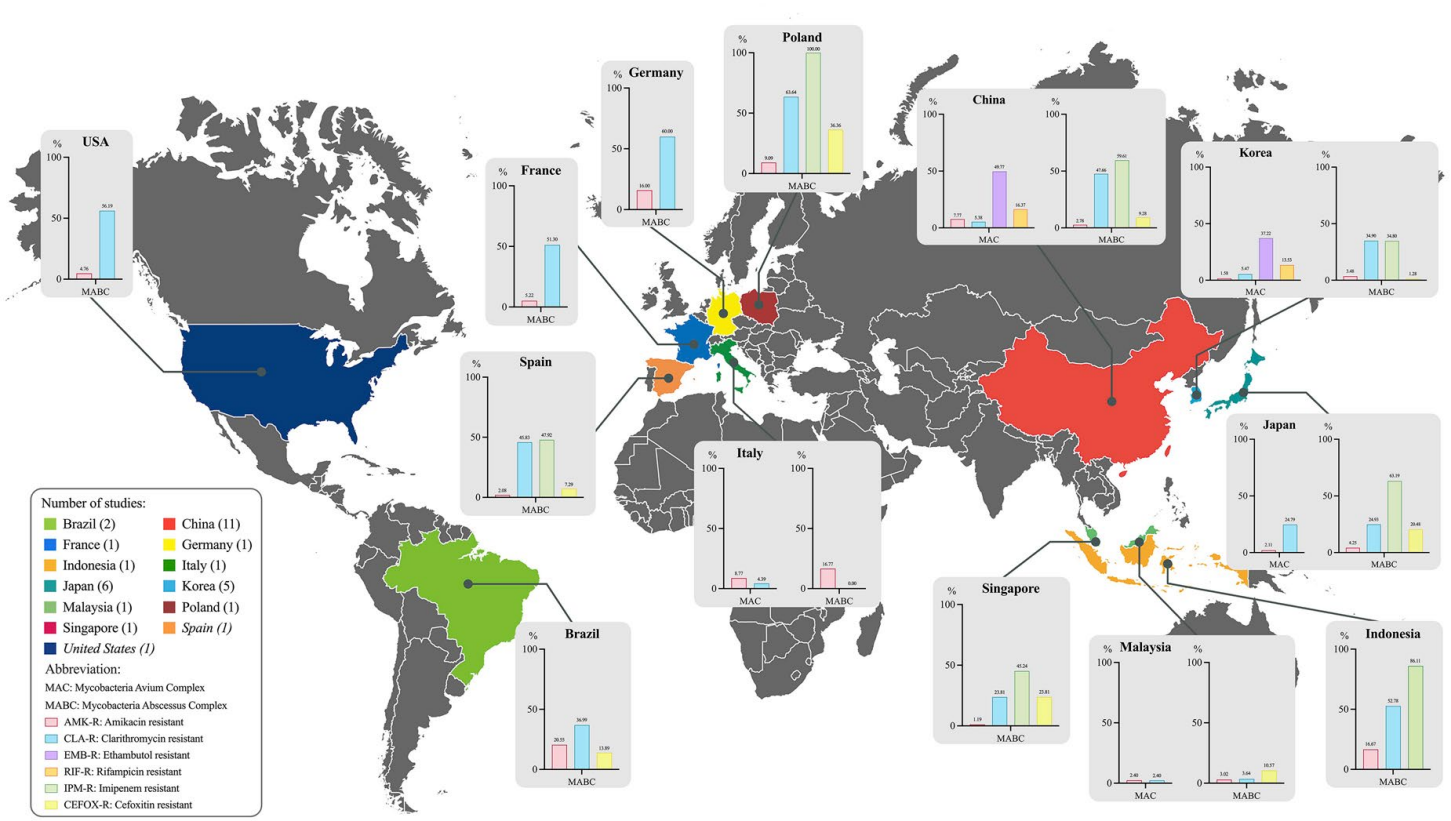
Antibiotic resistance situation of NTM.

In this part, a total of 33 studies from 13 countries were enrolled. The drug susceptibility results of six antibiotics were summarized. Amikacin (AMK) among Aminoglycosides and Clarithromycin (CLA) among Macrolides are first-line antibiotics for NTM therapy. Ethambutol (EMB) and Rifampicin (RIF) are commonly used for the standard treatment of Slowly Growing Mycobacteria (SGM) infection. As well as Imipenem (IPM) and Cefoxitin (CEFOX), which are more clinically relevant to Rapidly Growing Mycobacteria (RGM). All drug susceptibility results were interpreted uniformly based on the latest Antimicrobial Susceptibility Testing Standard from the Clinical and Laboratory Standards Institute (M100-2024). The NTM antibiotic resistance situation of each country is shown in Figure 5. Details of the drug susceptibility results for SGM and RGM were summarized in Tables 5 and 6, respectively.

**Table 5.** Drug susceptibility results of slowly growing mycobacteria (SGM).

Study	Region	Year	Species	Amikacin (*n* = 1978)			Clarithromycin (*n* = 2156)			Ethambutol (*n* = 506)			Rifampicin (*n* = 518)		
				R	I	S	R	I	S	R	I	S	R	I	S
G. Wei et al.	China	2015	MAV	1	9	40	1	2	47	49	1	0	15	0	35
W. Nie et al.	China	2015	MAC	–	–	–	6	1	124	–	–	–	–	–	–
			MKA	–	–	–	0	0	34	–	–	–	–	–	–
T. Hirama et al.	Japan	2016	MAV	–	–	–	10	0	9	–	–	–	–	–	–
			MIN	–	–	–	2	0	1	–	–	–	–	–	–
CC. Huang et al.	China TW	2017	MAV	0	2	6	0	0	8	–	–	–	–	–	–
			MIN	2	7	66	0	6	69	–	–	–	–	–	–
HJ. Huh et al.	Korea	2019	MAV	3	8	12	9	0	14	–	–	–	–	–	–
			MIN	1	15	19	16	0	9	–	–	–	–	–	–
CF. Liu et al.	China	2021	MAC	11	20	77	5	0	103	–	–	–	–	–	–
			MKA	0	1	30	0	0	31	–	–	–	2	0	29
Y. Li et al.	China	2022	MAV	3	8	41	2	0	50	–	–	–	–	–	–
			MCH	3	1	18	5	0	17	–	–	–	–	–	–
			MIN	22	24	119	8	0	157	–	–	–	–	–	–
G. He et al.	China	2022	MAC	7	2	34	7	1	35	30	11	1	–	–	–
			MKA	0	0	14	0	0	14	6	0	8	0	0	14
D. Wang et al.	China	2022	MIN	7	5	109	8	2	111	27	33	61	13	8	100
KJ. Kim et al.	Korea	2022	MAV	1	21	69	1	0	90	–	–	–	–	–	–
			MIN	0	26	191	0	0	217	–	–	–	–	–	–
			MKA	0	0	23	0	0	23	–	–	–	1	0	22
J. Wang et al.	China	2023	MAC	6	16	132	8	3	143	–	–	–	–	–	–
K. Fukushima et al.	Japan	2023	MAV	2	2	63	13	1	54	–	–	–	–	–	–
			MCH	0	0	2	1	0	1	–	–	–	–	–	–
			MIN	0	0	25	3	1	21	–	–	–	–	–	–
M. Kim et al.	Korea	2023	MAV	3	30	99	2	0	130	76	42	14	17	40	75
			MIN	2	12	120	6	2	126	23	65	46	19	37	78
			MKA	1	0	12	0	0	13	1	1	11	1	0	12
A. Mazzarelli et al.	Italy	2024	MAV	3	12	45	1	3	56	–	–	–	–	–	–
			MCH	4	7	19	0	0	30	–	–	–	–	–	–
			MIN	3	3	18	4	0	20	–	–	–	–	–	–
			MKA	1	2	6	0	0	9	–	–	–	–	–	–
M. Mohamad Azranyi et al.	Malaysia	2024	MAV	1	14	155	3	10	157	–	–	–	–	–	–
			MIN	5	7	68	3	7	70	–	–	–	–	–	–

MAC: Mycobacterium Avium Complex, MAV: *Mycobacterium avium*, MCH: *Mycobacterium chimaera*, MIN: *Mycobacterium intracellulare*.

MKA: *Mycobacterium kansasii*; R: resistant, I: intermediate, S: susceptible.

**Table 6.** Drug susceptibility results of rapidly growing mycobacteria (RGM).

Study	Region	Year	Species	Amikacin (*n* = 2352)			Clarithromycin (*n* = 2161)			Imipenem (*n* = 1280)			Cefoxitin (*n* = 1367)		
				R	I	S	R	I	S	R	I	S	R	I	S
WJ. Koh et al.	Korea	2011	MABS	3	5	56	22	3	39	27	24	11	0	32	32
			MMAS	6	13	60	3	0	76	50	18	7	1	36	42
T. Harada et al.	Japan	2012	MABS	4	3	56	10	7	46	36	16	11	–	–	–
			MMAS	0	1	22	1	1	21	17	5	1	–	–	–
SP. Tseng et al.	China TW	2013	MABS	1	5	58	45	1	18	25	39	0	4	60	0
			MMAS	1	9	54	2	1	61	21	43	0	8	56	0
S. Yoshida et al.	Japan	2013	MABS	6	3	81	14	6	70	48	28	14	–	–	–
			MBOL	1	1	51	4	1	48	31	18	4	–	–	–
LS. Nunes et al.	Brazil	2014	MBOL	0	0	43	6	0	37	–	–	–	0	12	31
PH. Cândido et al.	Brazil	2014	MABS	1	2	3	4	1	1	–	–	–	2	4	0
			MBOL	14	0	10	17	0	7	–	–	–	8	15	0
W. Nie et al.	China	2014	MABS	0	1	44	37	3	5	3	9	33	0	24	21
			MBOL	1	0	24	1	0	24	4	4	17	1	18	6
SY. Kim et al.	Korea	2014	MABS	0	3	31	31	0	4	–	–	–	0	13	20
			MMAS	0	0	25	0	2	24	–	–	–	0	18	6
J. Kehrmann et al.	Germany	2016	MABS	4	5	19	23	0	5	–	–	–	–	–	–
			MBOL	0	0	3	3	0	0	–	–	–	–	–	–
			MMAS	4	0	15	4	0	15	–	–	–	–	–	–
KL. Chew et al.	Singapore	2017	MABS	0	3	17	16	0	4	8	11	1	2	18	0
			MBOL	0	0	5	3	0	2	2	3	0	2	3	0
			MMAS	1	5	53	1	0	58	28	30	1	16	43	0
A. Aono et al.	Japan	2018	MABS	3	35	10	45	1	2	41	7	0	8	38	2
			MMAS	1	20	14	1	0	34	34	1	0	9	25	1
M. Wu et al.	China	2019	MABS	5	0	188	–	–	–	–	–	–	–	–	–
HJ. Huh et al.	Korea	2019	MABS	1	12	34	10	25	12	–	–	–	–	–	–
			MBOL	0	0	2	1	1	0	–	–	–	–	–	–
			MMAS	3	10	25	18	0	20	–	–	–	–	–	–
N. Bouzinbi et al.	France	2020	MABS	4	0	56	46	0	14	–	–	–	–	–	–
			MBOL	0	0	8	8	0	0	–	–	–	–	–	–
			MMAS	2	0	45	5	0	42	–	–	–	–	–	–
CF. Liu et al.	China	2021	MABS	4	1	62	56	2	9	67	0	0	–	–	–
			MBOL	0	1	1	1	0	1	–	–	–	–	–	–
			MMAS	0	0	45	5	1	39	44	1	0	–	–	–
S. Realegeno et al.	USA	2021	MABS	5	15	80	58	0	42	–	–	–	–	–	–
			MBOL	0	0	1	1	0	0	–	–	–	–	–	–
			MMAS	0	0	4	0	0	4	–	–	–	–	–	–
D. Wang et al.	China	2022	MABS	5	4	22	7	2	22	–	–	–	10	0	21
G. He et al.	China	2022	MABC	1	1	22	10	2	12	23	1	0	4	20	0
L. Saptawati et al.	Indonesia	2022	MABS	6	0	30	19	2	15	31	5	0	–	–	–
KJ. Kim et al.	Korea	2022	MABS	0	3	35	28	0	10	0	15	23	0	27	11
			MMAS	0	0	18	1	0	17	0	11	7	0	17	1
J. Wang et al.	China	2023	MABS	1	2	71	63	1	10	56	17	1	5	51	18
			MBOL	0	0	12	5	0	7	3	0	9	1	9	2
			MMAS	0	0	38	2	0	36	27	10	1	2	28	8
K. Fukushima et al.	Japan	2023	MABS	0	0	3	3	0	0	–	–	–	–	–	–
			MMAS	0	0	5	0	0	5	–	–	–	–	–	–
M. Kim et al.	Korea	2023	MABS	1	7	29	26	0	11	9	16	12	3	31	3
			MMAS	0	2	18	1	0	19	1	13	6	0	18	2
K. Kania et al.	Poland	2023	MABS	0	0	8	6	0	2	8	0	0	3	5	0
			MBOL	0	0	1	1	0	0	1	0	0	0	1	0
			MMAS	1	0	1	0	0	2	2	0	0	1	1	0
A. Ruedas-López et al.	Spain	2023	MABS	1	0	49	31	0	19	24	14	12	4	20	26
			MBOL	0	0	13	13	0	0	3	6	4	0	7	6
			MMAS	1	0	32	0	1	32	19	9	5	3	20	10
A. Mazzarelli et al.	Italy	2024	MABS	2	1	9	0	0	12	–	–	–	–	–	–
S. Takei et al.	Japan	2024	MABS	0	0	11	8	1	2	3	6	2	–	–	–
			MMAS	0	0	22	2	0	20	8	8	6	–	–	–
M. Mohamad Azranyi et al.	Malaysia	2024	MABC	10	24	297	12	7	312	–	–	–	35	270	26

MABC: Mycobacterium Abscessus Complex, MABS: *Mycobacterium abscessus*, MBOL: *Mycobacterium bolletii*, MMAS: *Mycobacterium massiliense*; R: resistant, I: intermediate, S: susceptible.

**Figure 6.**
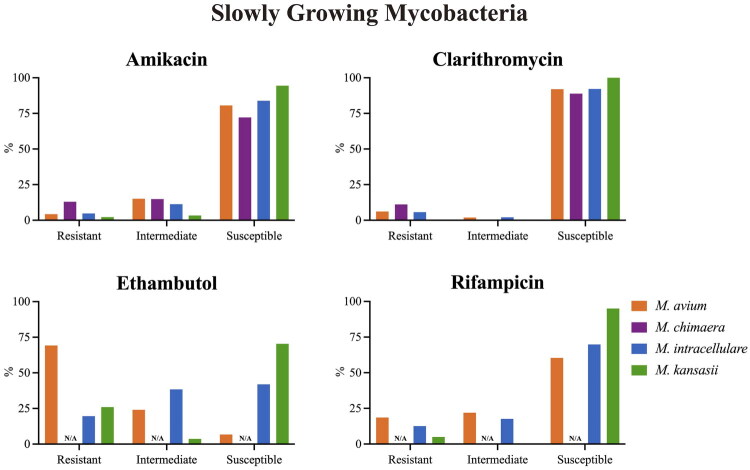
Comparison of SGM drug susceptibility among species.

**Figure 7.**
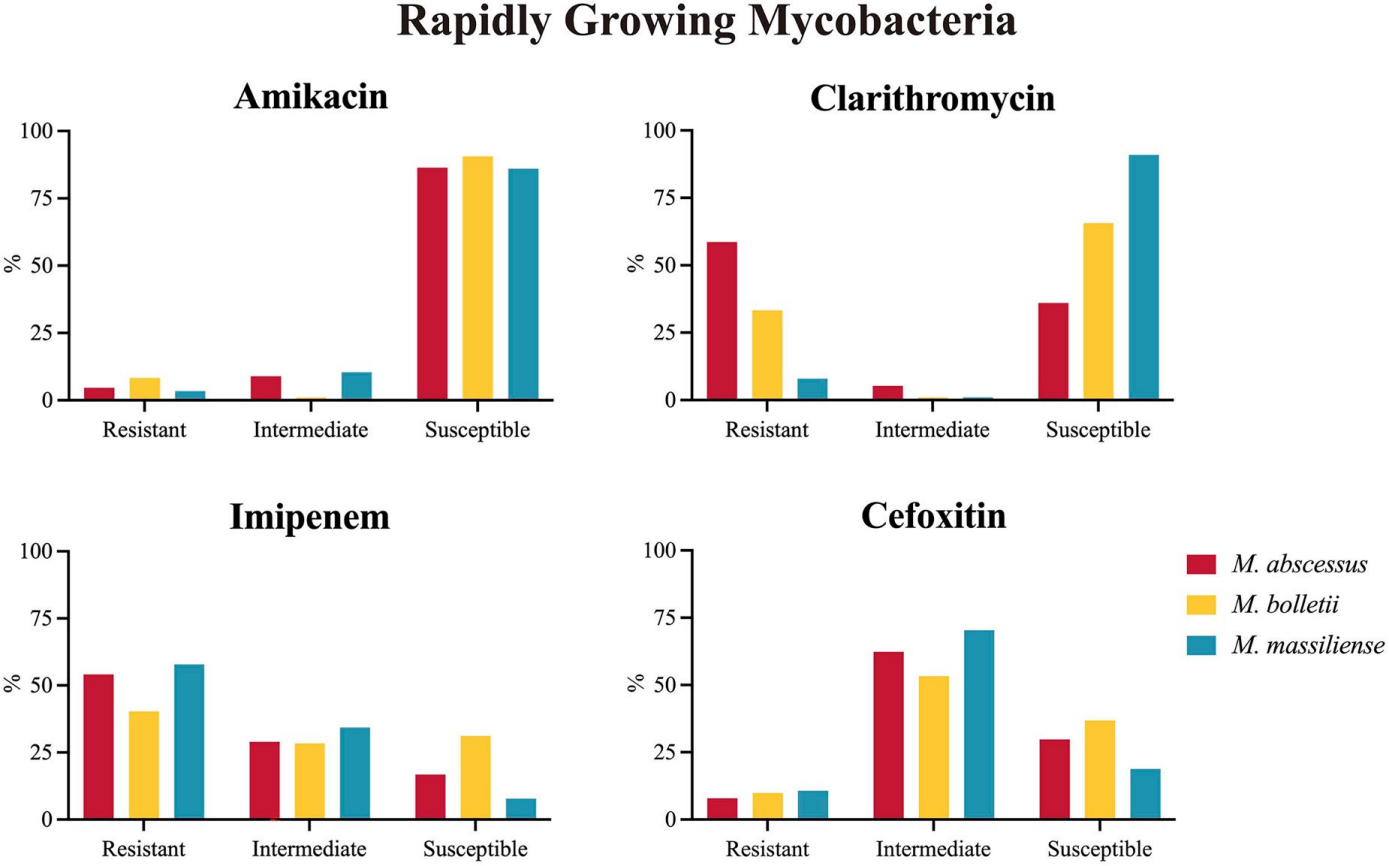
Comparison of RGM drug susceptibility among species.

For the SGM, the overall resistance rates for AMK, CLA, EMB and RIF were 4.65%, 5.75% 41.90% and 13.13%, respectively. As shown in Figure 6, the AMK resistance rate of *M. chimaera* (12.96%) was significantly higher than *M. avium* (4.28%), *M. intracellulare* (4.80%) and *M. kansasii* (2.22%) (*p* < 0.05). The CLA resistance rates of *M. kansasii* (0%) were lower than those of the three species in MAC (*p* < 0.01). The EMB resistance rate of *M. avium* (69.20%) was significantly higher than *M. intracellulare* (19.61%) and *M. kansasii* (25.93%) (*p* < 0.05). Besides, the RIF resistance rate of *M. kansasii* (4.94%) was also lower than *M. avium* (18.58%) and *M. intracellulare* (12.55%). For the RGM, the overall resistance rates for AMK, CLA, IPM and CEFOX were 4.66%, 39.76%, 54.22% and 9.19%, respectively. However, as shown in Figure 7, there was a different resistance rate among the subspecies of Mycobacterium Abscessus Complex (MABC). The AMK resistance rate of *M. bolletii* (8.33%) is slightly higher than that of *M. abscessus* (4.64%) and *M. massiliense* (3.47%) (*p* < 0.05). For another antibiotic, CLA, 58.63% of *M. abscessus* were resistant to CLA, which was significantly higher than *M. bolletii* (33.33%) and *M. massiliense* (7.97%) (*p* < 0.01). Regarding IPM and CEFOX resistance, the differences among subspecies were not statistically significant. Further details of drug susceptibility are presented in Supplementary File 4.

## Discussion

This systematic review and meta-analysis provide a comprehensive evaluation of emerging molecular technologies for NTM species or subspecies identification, along with antibiotic resistance profiling. Our findings underscore that significant advancements have been achieved in NTM identification capabilities with the application of these techniques, which subsequently benefit the precise treatment of NTM PD. However, the alarming situation of the NTM antibiotic resistance all around the world is challenging to the selection of the therapy regimen.

As the most widely adopted molecular technique for NTM identification, MALDI-TOF MS demonstrates good performance with a synthetic sensitivity of 0.92 (95% CI: 0.87, 0.96) at the species level. Compared to the traditional biochemical method, it not only prevails in accuracy but also has the advantage of fast testing and high throughput. The pre-processing steps and testing on the machine are relatively simple, which means the possibility of multi-sample testing in a short time [106]. Currently, Bruker, BioMérieux and some other companies have provided commercialized solutions for bacterial strain identification based on MALDI-TOF with a reasonable price on consumables. On the other hand, the identifying efficacy could also be improved as the software being updated. The identification sensitivity is significantly higher with the latest version of the database and identification algorithm [19,22,33]. Furthermore, with the application of user-customized algorithms or machine learning, even conclusive identification at the subspecies level is available [66,73]. Despite the advantages above, MALDI-TOF identification is still culture-based. Clinical specimens are not suitable for direct identification due to the richness in other proteins from humans that might interfere with mass spectra. Besides, our result also finds a difference in performance between solid and liquid medium that colony from the solid medium has better identification sensitivity. Similarly, lower sensitivity using colony from liquid medium was observed in the studies from Quinlan et al. and Lotz et al. [32,107]. They attributed the lower numbers of organisms in the liquid medium as the reason for lower efficacy. But according to another research, the impact on the obtained peaks in mass and intensity from different nutrients led to divergence of efficacy [108]. Although the exact mechanism needs further investigation, it suggests that the priority of colony from solid medium should be higher than from liquid medium in MALDI-TOF identification.

As researchers’ second favourite molecular method, the combined effect size is even better than MALDI, 0.98 (95% CI: 0.97, 0.99) at the species level. Further considering the penetration rate of PCR instruments in microbiology laboratories, PCR-based methods might be the most cost-effective choice for NTM identification. Firstly, the cost of instruments is lower than that of mass spectrum-based and sequencing-based technologies. For fluorescent probe-based High-Resolution Melting Analysis (HRMA), a Fluorescence quantitative Real-time PCR instrument is required, but its price is relatively lower [18,38,39,41,43,48]. Secondly, no expensive consumables are needed for PCR-based methods. Methods based on PCR-Reverse Blot Hybridization Assay (PCR-REBA) and PCR-GenoBlot assay have commercial kits for NTM identification at a reasonable price [40,44]. PCR Restriction enzyme Analysis (PRA) could also provide a low cost [42,49]. Thirdly, an equal performance using the clinical specimen could contribute to the timeliness. But limitation exists as well, the identification accuracy decreased as the presence rate of acid-fast bacilli in the smear decreased [109]. Our previous study also found a significantly higher accuracy and shorter Turnaround Time (TAT) in smear-positive samples when identifying Tuberculosis with PCR [16]. These remind us that if we want to shorten TAT by using clinical specimen testing, an effective sampling is the key as well. About subspecies identification, the PCR-based method excels in distinguishing subspecies of MABC [69]. But for the two subspecies of *M. intracellulare*, high sensitivity couldn’t be achieved at the same time [18,39]. We assume that the high sequence similarity of *M. intracellulare* and *M. chimaera* (over 99%) makes primer design and other experimental parameters setting difficult.

Among other techniques applied to NTM species identification, sequencing-based methods have achieved the highest synthetic sensitivity of 0.99 (95% CI: 0.98, 1.00). In fact, every sample (*n* = 252) in the included studies was successfully identified regardless of the sequencing platform and sample type used [53–55]. By sequencing, not only conclusive identification of NTM species/subspecies was delivered, but the discovery of Antibiotic Resistance Genes (ARGs) utilizing database matching and further ARGs analysis through Bioinformatics was also enabled [110]. About the shortcomings of sequencing-based techniques, it must be the expensive instruments and consumables, which make it difficult to apply on a wide scale. DNA chip technology showed excellent synthetic sensitivity (0.99, 95% CI: 0.80, 1.00) as well, particularly effective with the culture colony [57–60]. A significantly reduced sensitivity (0.692) with direct clinical specimens implies that its efficacy might highly depend on sufficient DNA concentration in the sample [56]. The DNA strip assay was first commercially available for Mycobacterium tuberculosis complex detection in the early 2000s [111]. Subsequently, kits suitable for NTM identification were developed. The existing commercial kits for NTM performed well in either species or subspecies identification [22,45,56,61,67,72]. Other technologies based on Cas12a/guide RNA, Flow cytometry, Quantamatrix Multiplexed Assay platform, and Single Nucleotide Polymorphisms genotyping also showed great potential in NTM identification, with the sensitivity all above 0.970 [62–65].

The preliminary antibiotic resistance patterns summarized in this study, while highlighting a concerning landscape, must be interpreted with explicit acknowledgement of their inherent limitations. These data are derived from heterogeneous studies that primarily reported phenotypic susceptibility results, often lacking critical ancillary information such as patient treatment history, clinical outcomes, or differentiation of key resistance mechanisms (e.g. *erm* [41] C28 versus T28 sequevars in *M. abscessus*). These aggregated data, however, serve to underscore significant challenges in current NTM management. Our findings reveal a significantly different resistance rate to CLA among MABC, aligning with previous studies that emphasized the clinical importance of accurate subspecies classification for guiding therapy. The acquired resistance to macrolides is related to a mutation in *rrl* gene encoding the peptidyltransferase domain of 23S rRNA, while the inducible resistance is caused by the *erm* [41] gene [112]. *M. abscessus* subspecies *abscessus* carrying the intact *erm* [41] gene develops inducible resistance upon exposure to macrolide antibiotics. Conversely, *M. abscessus* subspecies *massiliense* typically lacks this mechanism due to inactivating mutations in the *erm* [41] gene, resulting in significantly lower resistance rates. Up-regulated expression of efflux pump genes is another crucial mechanism for acquired resistance, particularly to macrolides and quinolones. The combination of antibiotics and efflux pump inhibitors could improve SGM’s drug susceptibility [113,114]. Different from SGM, RGM’s resistance to quinolones has been attributed to the mutation of the *gyrA* and *gyrB* genes [115]. Most of the NTMs are still susceptible to Aminoglycosides. Nucleotide substitutions in the *rss* gene or a mutation in the *rspL* gene coding for ribosomal protein S12 would lead to resistance [116]. Regarding other antibiotics, the high rates of resistance to drugs like Ethambutol (41.90% in SGM) and Imipenem (54.22% in RGM) signal therapeutic challenges but also call for a deeper investigation into whether these phenotypes correlate with specific genetic mutations or treatment failures. Although mass spectrum-based and sequencing-based technologies have demonstrated their unique strengths in the detection of ARGs, phenotypic drug susceptibility tests remain irreplaceable [98,117]. An accurate estimation of Minimum Inhibitory Concentration (MIC) is still unavailable using these molecular techniques. Clinically, the confirmation of antibiotic type and effective dosage depends on the MIC. These challenges collectively strengthen the argument for integrating molecular diagnostics more deeply into the NTM diagnostic pathway, not only for identification but also for resistance prediction. Hopefully, with the advancement of technologies, we might achieve accurate MIC estimation by combining molecular techniques with machine learning or other bioinformatics methods soon.

## Conclusion

In summary, emerging molecular technologies have revolutionized the identification of NTM, offering a solution with both accuracy and timeliness for NTM identification, and subsequently benefit the precise treatment of NTM PD. Various susceptibilities to antibiotics among NTM species and the increasingly severe resistance situation also underscore the significance of precise NTM species identification and drug susceptibility results for rational clinical regimen and prognosis assessment.

## Supplementary Material

Supplementary File 2 Identification sensitivity for six pulmonary infection NTM.docx

Supplementary File 3 Summary of diagnostic performance for each identification method.docx

Supplementary File 1 QUADAS2 Q and A.xlsx

PRISMA_checklist.docx

Supplementary File 4 Comparison of Drug susceptibility among species.xlsx

## Data Availability

The data that support the findings of this study are available from the corresponding author upon reasonable request.
