# Supplementary material for: Comparative evaluation of molecular technologies for the identification of prevalent non-tuberculous mycobacteria in pulmonary infections: a systematic review and meta-analysis
Source: Ann Med. 2026 Feb 10;58(1):2626123. doi: 10.1080/07853890.2026.2626123 (PMC12895907; doi:10.1080/07853890.2026.2626123)
Supplement: Supplementary File 2 Identification sensitivity for six pulmonary infection NTM.docx [file IANN_A_2626123_SM3155.docx]

| Supplementary File 2. Identification sensitivity for six pulmonary infection NTM | | | | | | | | | | | | | |
| --- | --- | --- | --- | --- | --- | --- | --- | --- | --- | --- | --- | --- | --- |
| Study | Sample type | *M. avium* | | *M. chimaera* | | *M. intracellulare* | | *M. kansasii* | | *M. xenopi* | | *M. abscessus* | |
|  |  | No. | Sen | No. | Sen | No. | Sen | No. | Sen | No. | Sen | No. | Sen |
| A. Alcolea-Medina et al.^17^ | Solid | 20 | 0.600 | - | - | 33 | 0.818 | 3 | 1.000 | - | - | 26 | 0.923 |
| J. Guiraud et al.^18^ | Solid | 9 | 1.000 | 1 | 1.000 | 3 | 1.000 | 3 | 1.000 | 2 | 1.000 | 1 | 1.000 |
|  | Liquid | 11 | 0.909 | 4 | 1.000 | 14 | 0.929 | 3 | 1.000 | 6 | 0.500 | 10 | 0.800 |
| BA. Brown-Elliott et al.^19^ | Solid | 9 | 1.000 | 8 | 1.000 | 9 | 1.000 | 9 | 1.000 | 9 | 1.000 | 38 | 1.000 |
| M. Markanović et al.^20^ | Liquid | 25 | 1.000 | 2 | 1.000 | 18 | 1.000 | 2 | 1.000 | 23 | 1.000 | 2 | 1.000 |
| GE. Genc et al.^21^ | Liquid | 16 | 0.938 | - | - | 3 | 0.667 | 4 | 1.000 | 1 | 1.000 | 65 | 1.000 |
| B. Rodríguez-Sánchez et al.^22^ | Solid | 17 | 0.824 | - | - | 5 | 0.800 | 10 | 1.000 | 5 | 0.800 | 13 | 0.692 |
| I Mareković et al.^23^ | Liquid | 8 | 0.875 | - | - | 4 | 0.000 | - | - | 2 | 1.000 | 1 | 1.000 |
| Y. Zhu et al.^24^ | Solid | 20 | 0.900 | - | - | 500 | 0.992 | 51 | 0.980 | 1 | 1.000 | 69 | 1.000 |
| IY. Yoo et al.^25^ | Liquid | 40 | 0.875 | 1 | 1.000 | 60 | 0.850 | 1 | 1.000 | 1 | 0.000 | 43 | 0.907 |
|  | Subculture | 40 | 0.959 | 1 | 1.000 | 60 | 0.967 | 1 | 1.000 | 1 | 1.000 | 43 | 0.953 |
| I. Akyar et al.^26^ | Solid | 4 | 1.000 | 2 | 1.000 | 4 | 0.750 | 8 | 1.000 | - | - | 11 | 0.909 |
| L. Luo et al.^27^ | Solid | 56 | 0.964 | 1 | 0.000 | 153 | 0.980 | 68 | 0.971 | 2 | 1.000 | 90 | 0.978 |
| D. Rodríguez-Temporal et al.^28^ | Liquid | 12 | 0.917 | - | - | 13 | 0.846 | 5 | 1.000 | 3 | 1.000 | 18 | 1.000 |
| JY. Chien et al.^29^ | Liquid | 12 | 0.500 | - | - | 22 | 0.591 | 3 | 0.333 | - | - | 20 | 0.750 |
| G. Tudó et al.^30^ | Solid | 18 | 0.722 | - | - | 6 | 0.667 | - | - | - | - | 8 | 0.625 |
|  | Liquid | 9 | 0.333 | - | - | 4 | 1.000 | 1 | 0.000 | 3 | 0.667 | 7 | 0.857 |
| PJ. Ceyssens et al.^31^ | Solid | 13 | 1.000 | 5 | 0.600 | 5 | 1.000 | - | - | 5 | 1.000 | 1 | 1.000 |
| P. Quinlan et al.^32^ | Solid | 2 | 1.000 | - | - | 3 | 1.000 | 4 | 1.000 | - | - | 2 | 1.000 |
|  | Liquid | 10 | 0.500 | - | - | 3 | 0.333 | 4 | 1.000 | 1 | 1.000 | 5 | 1.000 |
| J. Kehrmann et al.^33^ | Solid | 23 | 1.000 | - | - | 12 | 0.583 | 12 | 0.333 | 9 | 0.333 | 33 | 1.000 |
|  | Liquid | 23 | 0.870 | - | - | 12 | 0.917 | 12 | 0.333 | 9 | 0.778 | 33 | 1.000 |
| E. Miller et al.^34^ | Solid | 15 | 1.000 | - | - | 13 | 0.850 | - | - | - | - | 1 | 1.000 |
|  | Liquid | 25 | 0.800 | - | - | 3 | 1.000 | - | - | - | - | - | - |
| E. Oliva et al.^35^ | Solid | 4 | 1.000 | - | - | 1 | 1.000 | 2 | 1.000 | 3 | 1.000 | 1 | 1.000 |
|  | Liquid | 4 | 0.750 | - | - | 1 | 1.000 | 2 | 1.000 | 3 | 1.000 | 1 | 1.000 |
| L. Luo et al.^36^ | Liquid | 75 | 0.800 | 2 | 1.000 | 271 | 0.827 | 111 | 0.793 | 7 | 0.286 | 284 | 0.891 |
| M. Kodana et al.^37^ | Liquid | 24 | 0.958 | - | - | 13 | 0.923 | 14 | 1.000 | - | - | 8 | 1.000 |
| J. Lin et al.^38^ | Liquid | 4 | 1.000 | - | - | 130 | 1.000 | 9 | 1.000 | - | - | 39 | 1.000 |
| B. Luukinen et al.^39^ | Solid | 9 | 1.000 | 3 | 1.000 | 2 | 0.000 | 3 | 1.000 | - | - | 4 | 1.000 |
|  | Liquid | 24 | 0.958 | 5 | 1.000 | 10 | 0.500 | 1 | 1.000 | 1 | 1.000 | 10 | 1.000 |
| SH. Kim et al.^40^ | Undeclared | 14 | 1.000 | - | - | 9 | 1.000 | 14 | 0.929 | - | - | 16 | 0.875 |
| ADS. Peixoto et al.^41^ | Liquid | 7 | 0.571 | - | - | - | - | 37 | 0.946 | - | - | 10 | 0.800 |
| Ö. Appak et al.^42^ | Solid | 2 | 1.000 | - | - | 6 | 0.833 | - | - | 26 | 1.000 | 71 | 1.000 |
| AD. Khosravi et al.^43^  Q. Zhang et al.^44^ | Solid | 5 | 1.000 | - | - | 5 | 0.600 | 5 | 1.000 | - | - | 5 | 1.000 |
|  | Clinical | 36 | 1.000 | - | - | 236 | 0.983 | 46 | 1.000 | 2 | 1.000 | 106 | 1.000 |
| M. Yang et al.^45^ | Mixed | 33 | 1.000 | - | - | 32 | 1.000 | - | - | - | - | 31 | 1.000 |
| HY. Wang et al.^46^ | Clinical | 22 | 1.000 | - | - | 29 | 1.000 | 1 | 1.000 | - | - | 35 | 1.000 |
| HY. Wang et al.^47^ | Liquid | 18 | 1.000 | - | - | 38 | 1.000 | 2 | 1.000 | - | - | 3 | 1.000 |
| KL. Leung et al.^48^ | Solid | 40 | 0.950 | - | - | 197 | 0.980 | 62 | 1.000 | - | - | - | - |
| W. Cheunoy et al.^49^ | Solid | 23 | 1.000 | - | - | 4 | 1.000 | 9 | 1.000 | - | - | 44 | 0.977 |
| HJ. Huh et al.^50^ | Mixed | 81 | 1.000 | - | - | 55 | 0.964 | 1 | 1.000 | - | - | 64 | 1.000 |
| Y. Uwamino et al.^51^ | Clinical | 41 | 0.976 | - | - | 25 | 0.880 | 1 | 1.000 | - | - | 17 | 1.000 |
|  | Liquid | 41 | 1.000 | - | - | 25 | 1.000 | 1 | 1.000 | - | - | 17 | 1.000 |
| CH. Cha et al.^52^ | Liquid | 181 | 0.989 | - | - | 242 | 0.992 | 4 | 0.250 | - | - | 109 | 1.000 |
| J. Guiraud et al.^18^ | Solid | 9 | 1.000 | 1 | 1.000 | 3 | 1.000 | 3 | 1.000 | 2 | 1.000 | 1 | 1.000 |
|  | Liquid | 11 | 1.000 | 4 | 1.000 | 14 | 0.786 | 3 | 1.000 | 6 | 0.667 | 10 | 1.000 |
| K. Fukushima et al.^53^  J. Wang et al.^54^ | Liquid | 68 | 1.000 | 2 | 1.000 | 25 | 1.000 | 1 | 1.000 | - | - | 8 | 1.000 |
|  | Clinical | 1 | 1.000 | - | - | 2 | 1.000 | 4 | 1.000 | - | - | 3 | 1.000 |
| X. Yu et al.^55^ | Solid | 8 | 1.000 | - | - | 69 | 1.000 | 22 | 1.000 | - | - | 39 | 1.000 |
| HU. Schildhaus et al.^56^ | Clinical | 6 | 1.000 | 7 | 0.714 | 1 | 0.000 | 3 | 0.333 | 2 | 1.000 | 7 | 0.571 |
| H. Fang et al.^57^ | Solid | 38 | 1.000 | - | - | 170 | 0.994 | 31 | 1.000 | - | - | 67 | 0.955 |
| J. Liu et al.^58^ | Solid | 15 | 1.000 | - | - | 32 | 1.000 | 11 | 1.000 | - | - | 47 | 1.000 |
| MR. Lee et al.^59^ | Mixed | 8 | 0.375 | - | - | 20 | 0.950 | 4 | 0.750 | - | - | 38 | 0.947 |
| Y. Pang et al.^60^ | Solid | 38 | 1.000 | - | - | 139 | 1.000 | 22 | 1.000 | 1 | 1.000 | 153 | 1.000 |
| M. Yang et al.^45^ | Mixed | 33 | 1.000 | - | - | 32 | 1.000 | - | - | - | - | 31 | 1.000 |
| B. Rodríguez-Sánchez et al.^22^ | Solid | 17 | 0.941 | - | - | 5 | 0.800 | 10 | 1.000 | 5 | 1.000 | 13 | 0.538 |
| HU. Schildhaus et al.^56^ | Clinical | 6 | 1.000 | 7 | 1.000 | 1 | 0.000 | 3 | 1.000 | 2 | 1.000 | 7 | 0.714 |
| AS. Lee et al.^61^ | Solid | 15 | 1.000 | - | - | 17 | 1.000 | 2 | 1.000 | 2 | 1.000 | 8 | 0.625 |
| G. Xiao et al.^62^ | Liquid | 10 | 1.000 | - | - | 15 | 0.933 | 7 | 1.000 | - | - | 15 | 1.000 |
| H. Li et al.^63^ | Liquid | 84 | 0.988 | - | - | 54 | 0.981 | 19 | 1.000 | - | - | 17 | 0.882 |
| HY. Wang et al.^64^ | Mixed | 17 | 1.000 | - | - | 34 | 0.971 | 2 | 1.000 | - | - | 15 | 0.933 |
| H. Wang et al.^65^ | Solid | 41 | 1.000 | - | - | 68 | 1.000 | 38 | 0.947 | - | - | - | - |
