# Supplementary material for: Comparative evaluation of molecular technologies for the identification of prevalent non-tuberculous mycobacteria in pulmonary infections: a systematic review and meta-analysis
Source: Ann Med. 2026 Feb 10;58(1):2626123. doi: 10.1080/07853890.2026.2626123 (PMC12895907; doi:10.1080/07853890.2026.2626123)
Supplement: Supplementary File 3 Summary of diagnostic performance for each identification method.docx [file IANN_A_2626123_SM3154.docx]

| Supplementary File 3. Summary of diagnostic performance for each identification method | | | |
| --- | --- | --- | --- |
| Identification Method | Study No. | Total sample No. | Sensitivity |
| MALDI-TOF Mass Spectrum | 21 | 2936 | 0.92 |
| PCR | 16 | 2425 | 0.98 |
| Sequencing | 3 | 252 | 0.99 |
| DNA chip | 5 | 860 | 0.99 |
| DNA strip (based on line probe assay) | 4 | 216 | 0.92 |
| Cas12a/gRNA | 1 | 47 | 0.98 |
| Flow cytometry | 1 | 174 | 0.98 |
| Quantamatrix | 1 | 68 | 0.97 |
| SNP genotyping | 1 | 147 | 0.99 |
